# Correlation Between Bone Mineral Density And Different Types of Modic Changes in Lumbar Spine

**DOI:** 10.2174/0115734056368021250428060738

**Published:** 2025-05-13

**Authors:** Xiaoling Zhong, Yinghui Tang, Guohua Zeng, Lixiang Zhang, Minjie Yang, Yu Chen

**Affiliations:** 1 Department of Radiology, Shenzhen People's Hospital (The First Affiliated Hospital of Southern University of Science and Technology, The Second Clinical Medical College of Jinan University), Shenzhen, Guangdong, China

**Keywords:** Bone mineral density, Lumbar spine, Modic change, Magnetic resonance imaging, Computed tomography, Modic changes, Lumbar Degenerative Disease

## Abstract

**Introduction:**

Modic changes (MCs) are a common manifestation of lumbar degenerative disease, classified into three types. However, the relationship between Bone Mineral Density (BMD) and each type of MC at the vertebral lesion sites remains unclear.

**Methods:**

This study included 144 patients who had both lumbar MR and CT images. The classification and grading of MCs were evaluated using MR images. On the CT images, BMD values, T-scores, and Z-scores were obtained from the normal T12 vertebrae, the corresponding lumbar Modic lesion sites, and the adjacent healthy regions at the same vertebra on the axial plane.

**Results:**

A total of 370 vertebrae (226 MCs and 144 normal T12 vertebrae) were assessed. No significant difference was found in the BMD of normal T12 vertebrae between males and females in the study. MCs were more commonly found in the lumbar 4 and 5 vertebrae. Of the MCs, 80 (36%) were classified as type I, 130 (57%) as type II, and 16 (7%) as type III. The BMD value, T-score, and Z-score of each Modic type lesion site were higher than those of adjacent healthy regions and normal T12 vertebrae. A strong correlation was found between the different Modic types, though no significant differences were observed between grades within the same Modic type.

**Conclusion:**

The presence of any MCs was significantly associated with an increase in BMD in the corresponding lesion sites, with more severe MCs showing a stronger association with higher BMD. This is the first study to explore the relationship between all types of MCs and their BMD values.

## 1 INTRODUCTION

Modic changes (MCs), proposed by Modic *et al*. in 1988, are a common manifestation of lumbar degenerative disease [1, 2]. Various factors can lead to the destruction of the endplate microstructure, resulting in vertebral marrow edema and fibrosis, which gradually leads to vertebral fat infiltration and, eventually, sclerosis of the vertebral body [3, 4].

Lumbar MCs are present in the general population, not just in patients with osteoporosis. The prevalence of MCs varies significantly across different studies [ 5 ].

Most studies have focused on the association between Bone Mineral Density (BMD) and Lumbar Degenerative Disease (LDD) in either the lumbar spine or femoral neck [6-9]. Less is known about the relationship between BMD and each type of MC in the vertebral lesion sites. Dual-energy X-ray Absorptiometry (DXA) is the gold standard for the diagnosis of osteoporosis, which calculates BMD. However, the accuracy of DXA may be limited by the presence of degenerative spinal pathology. In recent years, there has been growing interest in using alternative imaging, including CT and MRI, to assess BMD. These other modalities have potential advantages over DXA imaging. For example, studies have revealed that DXA imaging was less accurate than the CT Hounsfield unit (HU) in calculating BMD in patients with spinal degeneration [10]. Therefore, the purpose of the present study was to investigate the correlation between each Modic type in the lumbar spine and BMD using conventional CT, aiming to explain changes in the pathological process and provide valuable insights for clinical diagnosis.

## 2 MATERIALS AND METHODS

### 2.1 Study Population

This study was approved by the institutional ethics board. We retrospectively analyzed the data of consecutive patients from January 2022 to April 2024 at our radiology department PACS system. Patients with both lumbar MR images and CT scans (within one month) were eligible for inclusion. Exclusion criteria included age <18 or >80 years, history of lumbar vertebral fractures or surgery, other lumbar diseases or conditions affecting bone metabolism, undiagnosable or unmeasurable scan images, and use of bone metabolism medications. A total of 144 patients were included: 68 males and 76 females, with a mean age of 60.0 ± 10.7 years (range: 30 to 79 years). The participant selection process is illustrated in Fig. (**1**).

### 2.2 MR and CT Examination

Lumbar MR examinations were conducted using 1.5T or 3.0T MR scanners. Details of the MR image acquisition process are provided in the data supplement. Lumbar CT examinations were performed with the United Imaging uCT760 (United Imaging, China), using a tube voltage of 120 kV, automatic tube current, a slice thickness of 0.6 mm, a rotation time of 0.8 s, and a standard reconstruction algorithm. The lumbar CT scanning range extended from the upper edge of the Thoracic 12 vertebra (T12) to the lower edge of the Sacral 3 vertebra (S3). The MR and CT image data were then transferred to the PACS system and the Philips IntelliPortal workstation for further analysis.

### 2.3 Classification and Grade of Modic Changes

**Fig. (2):**
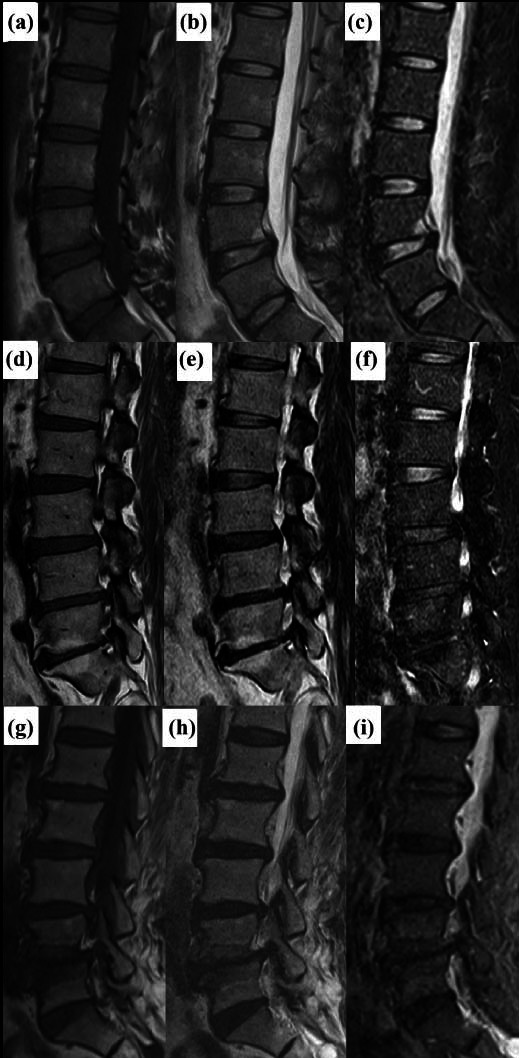
Classification and grade of MCs. (**a-c**/) Modic I, the endplate and adjacent bone showed low signal on T1WI, high signal on T2WI, and high signal on fat suppression, grade B (25%~50% of vertebral height ). (**d-f**) Modic II, high signal on T1WI, slightly high or equal signal on T2WI, and low signal on fat suppression, grade B. (**g-i**) Modic III, low signal on T1WI and T2WI, and low signal on fat suppression, grade B.

The classification was performed based on the studies by Modic *et al.* [1, 2]: Type I (edema type, the endplate, and adjacent bone, which showed low signal on T1WI, high signal on T2WI, and high signal on fat suppression); Type II (fat type, which showed high signal on T1WI, slightly high or equal signal on T2WI, and low signal on fat suppression); and Type III (sclerosis type, which showed low signal on T1WI and T2WI, and low signal on fat suppression). The severity of MCs was graded according to the studies by Weishaupt *et al.* and Udby *et al.* [11, 12]: Grade A (≤25% of vertebral height), considered mild degeneration; Grade B (25%~50% of vertebral height), considered moderate degeneration; and Grade C (≥50% of vertebral height), considered severe degeneration. The evaluations were performed by two board-certified radiologists independently without knowledge of the patient's history and clinical manifestations (Fig. **2**). If there was any disagreement, consensus was reached after discussion.

### 2.4 Bone Mineral Density Measurement

**Fig. (3):**
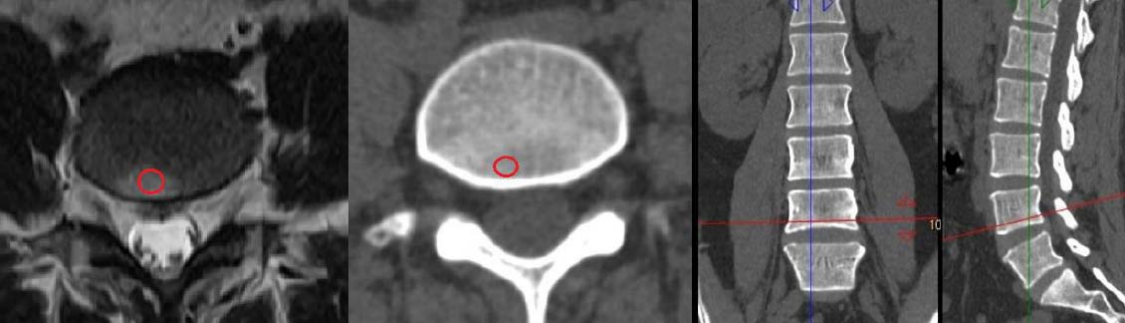
Example for BMD measurement in the MCs lesion (axial, sagittal and coronal).

BMD measurement was performed using the Philips IntelliSpace Portal workstation's Bone Mineral Density tool, which provides three-dimensional reconstructed CT images. The normal T12 vertebra, the corresponding lumbar Modic lesion sites, and adjacent healthy regions at the same vertebra on the axial plane were selected for analysis. The BMD value was measured by placing an oval region of interest (ROI) over the selected area. During manual ROI drawing, cortical bone and abnormal bone areas, such as bone islands and venous sinuses, were excluded (Fig. **3**). BMD measurements were conducted by two board-certified radiologists. Three measurements were taken for each patient, and the average BMD value was calculated to represent the bone density of the selected area. The BMD unit is mg/cm^3^.

### 2.5 Statistical Analysis

SPSS software (IBM SPSS Statistics for Windows, Version 25.0 IBM Corp, Armonk, NY, USA) was used for statistical analysis. Descriptive statistics, such as mean with standard deviation, were used for continuous data. Independent *t*-tests, Wilcoxon rank-sum tests, or Mann-Whitney tests as appropriate were used to compare continuous variables, while the χ^2^ tests were used to compare categorical data. An independent sample t-test was used between T12 vertebra and adjacent healthy regions of MCs. One-way ANOVA and Welch's test were used among groups. The games-Howell test was used for multiple comparisons. Spearman correlation was used to examine the correlation between MCs and BMD value, T-score, and Z-score. A *P*-value <0.05 was considered statistically significant.

## 3 RESULTS

Of 144 patients who met the inclusion criteria, there were a total of 370 vertebrae (226 MCs and 144 normal T12 vertebrae). No significant difference was found in the BMD of normal T12 vertebrae between men and females in the study. The distributions of Modic type and grade are shown in Fig. (**4**) and Table **1**. Mixed types of MCs were excluded (*i.excluded*., mixed I-II type, mixed II-III type). The distributions of MCs in lumbar 1-5 vertebrae are represented in Fig. (**5**).

The BMD value, T-score, and Z-score of the 370 vertebrae are shown in Table **2**. One-way ANOVA revealed significant statistical differences between groups (P < 0.001), and the subsequent Games-Howell test indicated significant differences between each pair (P < 0.001), except for the adjacent healthy region and normal T12 vertebra. Specifically, the BMD value, T-score, and Z-score of each Modic type lesion site were higher than those of the adjacent healthy regions and normal T12 vertebrae, with no significant difference observed between the adjacent healthy regions and normal T12 vertebrae.

There was a strong correlation between each Modic type (r=0.7, *P*<0.001); that is, the BMD of early Modic changes was smaller, and as the disease progressed, the BMD of advanced MCs increased. No significant difference was found between grades in the same Modic type.

No significant differences were found between women and men regarding Modic I and Modic III. However, there was a significant difference in the average BMD of Modic II between women and men (P = 0.001), with the average BMD of Modic II in women being slightly lower than that in men.

## 4 DISCUSSION

Our study demonstrated that the presence of any MCs was significantly associated with an increase in BMD at the corresponding lesion sites. Additionally, a trend was observed, where more severe MCs were strongly associated with higher BMD. In contrast, no significant association was found between the normal bone areas and between grades in the same Modic type.

As is well known, in 1988, Modic classified changes in vertebral marrow adjacent to the endplate into three types [1, 2]. Type I shows decreased signal intensity on T1WI and increased signal intensity on T2WI, indicating edema and vascularized fibrous tissues, which cause acute low back pain. Type II shows increased signal intensity on T1WI and T2WI, histopathologically representing yellow marrow replacement, and is thought to be a stable form. Type III is characterized by decreased signal intensity on T1WI and T2WI, corresponding to sclerosis of the endplate adjacent bone marrow. A variety of studies reported an association between chronic back pain and MCs, especially for acute Modic type I changes. The incidence rate of MCs is approximately 20% to 58% [5, 13, 14]. The prevalence of MCs in the present study was consistent with the published literature [2, 15]; that is, type II (57%) was the most common, with sclerotic type III being rare (7%).

Moreover, MCs are most commonly found at the L4 and L5 vertebrae rather than at L1-L3 [14, 16].

A longitudinal study tracing the evolution of type I change discovered that it is a dynamic process that either converts to type II change or becomes more extensive in a large majority of cases [17]. Kuisma *et al.* demonstrated that endplate sclerosis exists in all types of MCs [18]. Feng *et al.* revealed that the signal of most type II was not suppressed on fat suppression MR images, suggesting that there may be ongoing complicated pathologies [19]. Type II may not be merely a fat replacement. Sun *et al.* quantitatively measured the CT values of type II and found them significantly higher than those of non-Modic sites, implying that osteosclerosis may exist in type II [20]. The more severe the degeneration of the adjacent disc, the greater the degree of osteosclerosis. Okano *et al.* demonstrated that all types of MCs had independent positive effects on volumetric BMD [6]. Our results are in accordance with these prior studies. The increased BMD may reflect a pathological process in the marrow with new bone formation. In other anatomical research, Saadat *et al.* correlated MR imaging findings typically associated with osteoarthritis, such as bone marrow edema pattern and cartilage swelling, with histological findings [21]. They found that all cases of bone marrow edema on MRI subchondral ingrowth of fibrovascular tissue and increased bone remodeling were observed. Moreover, Lowitz *et al.* examined that BMD was significantly increased in bone marrow lesions in the knee [22].

Several studies have reported the relationships between vertebral BMD and LDD using different measurements. Miyakoshi *et al.* found that spondylotic changes in postmenopausal women exhibited positive correlations not only with the lumbar BMD but also with the remote-site BMD measured by Dual-energy X-Ray Absorptiometry (DXA) [23]. Using a micro-computed tomography system, Wang *et al.* clarified the association between vertebral BMD and disk degeneration and specifically indicated that it was higher BMD of the vertebral body, not the entire vertebra, that was associated with more severe adjacent disk degeneration [7]. Livshits *et al.* found that there was a significant positive correlation between LDD and BMD at the lumbar spine and hip [8]. Both traits were highly heritable, and the associations between them were mediated genetically. Okano *et al.* demonstrated that MCs, higher total end plate score, and vacuum phenomenon were significantly associated with an increase in regional trabecular vBMDs measured by quantitative CT in apparently normal bone areas [6]. However, Margulies *et al.* indicated that subjects with severe osteoporosis in the distal radius also had severe degenerative changes in the discs and the facets, whereas those with mild osteoporosis in the distal radius showed a tendency to have a lesser degree of degenerative changes [24]. Grams *et al.* found that degenerative spinal changes may be associated with reduced regional spinal mineralization using quantitative CT [8]. In our study, we used a simple yet feasible method to investigate the correlation between each Modic type in the lumbar spine and its BMD, a method that can be easily implemented by average hospital staff. Interestingly, our study found no significant difference in BMD between normal bone marrow areas or between grades within the same Modic type.

Nonetheless, our study has several limitations. Due to the retrospective study design and small sample size, these findings need to be confirmed in further prospective studies. Second, MRI scans in the present study were performed in different machines due to clinical indications for imaging, so MRI detection signal bias should not be neglected. However, MR imaging was acquired at a standard protocol, which was internationally recognized. The classification of Modic changes has already been shown to be reliable and reproducible. Finally, these radiograph investigations should be histologically confirmed. Despite these shortcomings, we believe that our findings are of importance for the accurate diagnosis and management of MCs.

## CONCLUSION

Our results demonstrated that the presence of any Modic changes was significantly associated with increased BMD at the corresponding lesion sites. Additionally, a trend was observed where more severe Modic changes were strongly associated with higher BMD. The clinical significance of these findings is that there may be sclerotic changes in all types of Modic changes. However, no significant association was found between the normal bone areas, suggesting that BMD changes in the entire lumbar spine may not be directly caused.

## Figures and Tables

**Fig. (1):**
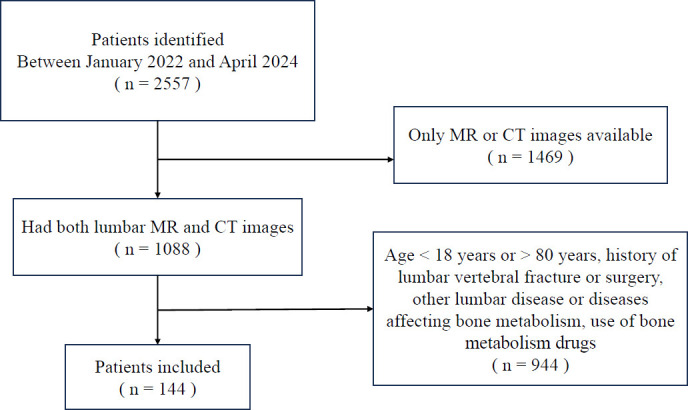
Study flow diagram.

**Fig. (4):**
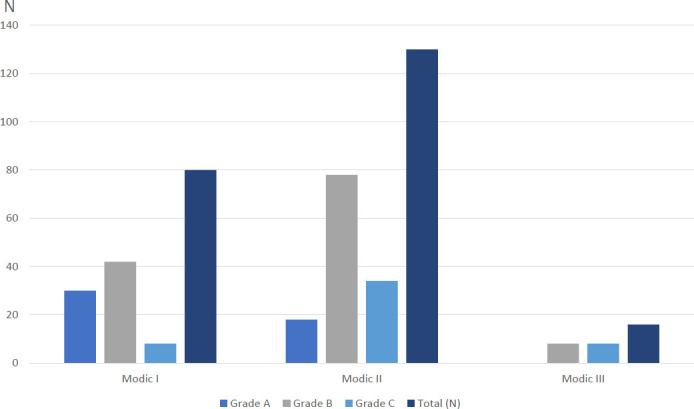
Distribution of Modic types. Type II was most common (57%) with the sclerotic type III being rare (7%).

**Fig. (5):**
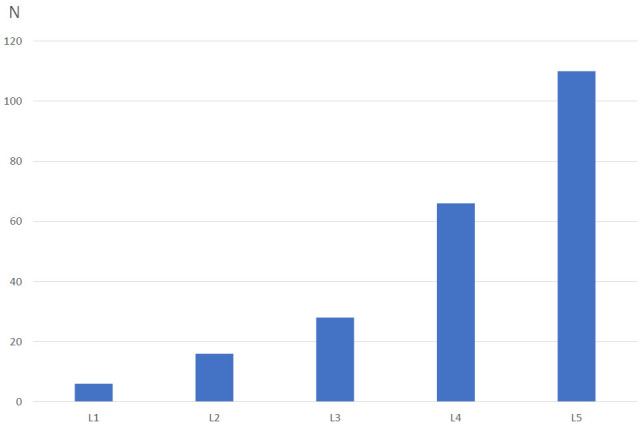
Distribution of MCs in vertebra. MCs were found to be more common at L4-5 rather than L1-3.

**Table 1:** Distributions of Modic types and grades.

	Grade A	Grade B	Grade C	Total (N)
Modic I	30	42	8	80
Modic II	18	78	34	130
Modic III	0	8	8	16
total	48	128	50	226

**Table 2:** Comparisons of BMD value, T-score, and Z-score in different groups.

	Modic I	Modic II	Modic III	Adjacent healthy region	T12 vertebra	*P* value
BMD value	164.35 ± 59.94	218.83 ± 67.19	302.38 ± 21.73	77.90 ± 34.58	85.11 ± 31.49	**0.000**
T value	-0.62 ± 1.89	1.18 ± 2.13	3.83 ± 0.72	-3.33 ± 1.09	-3.11 ± 0.98	**0.000**
Z value	1.11 ± 1.86	3.31 ± 2.08	6.15 ± 1.22	-1.13 ± 0.80	-0.87 ± 0.74	**0.000**

Date are presented in mean ± SD. One-way ANOVA showed that there were significant statistical differences between groups (*P*<0.001), and further Games-Howell test demonstrated that there were significant statistical differences between each pair (*P*<0.001) except for adjacent healthy region and normal T12 vertebrae. *P*<0.05 is considered statistically significant indicated in bold.

## Data Availability

All the data and supporting information are provided within the article.

## LIST OF ABBREVIATIONS

BMD: Bone Mineral Density
MCs: Modic Changes
HU: Hounsfield Unit
ROI: Region of Interest

## STANDARDS OF REPORTING

STROBE guidelines were followed.
